# Proof-of-concept validation of p2GUS, a modular expression system for enhanced Ni-binding purification of functional recombinant proteins in Escherichia coli

**DOI:** 10.1007/s00253-026-13968-x

**Published:** 2026-08-08

**Authors:** Pedro Ferro-Gallego, Lourdes Domínguez-Gerpe

**Affiliations:** 1 Department of Biochemistry and Molecular Biology, University of Santiago de Compostela, Santiago de Compostela, 15782 Spain; 2 IBIMA, Instituto de Investigación Biomédica y Plataforma en Nanomedicina, BIONAND, Málaga, 29590 Spain

**Keywords:** p2GUS expression system, Tandem His₁₀ tags, Multivalent affinity architecture, Seamless cloning, Recombinant protein purification, Post-expression processing, DNA-binding proteins

## Abstract

**Abstract:**

Protein expression and purification remain critical steps in basic research, biotechnology, and industrial applications. Here, p2GUS, a modular *Escherichia coli* expression system generated through targeted modification of Precursor 1, a parental pBAD-derived vector, is described. The principal feature is a tandem His₁₀–GST–His₁₀ architecture designed to enhance interaction with Ni–NTA matrices while retaining compatibility with post-expression processing strategies. The GST module serves primarily as a spacer between the two polyhistidine tags while also contributing solubility-enhancing properties. Engineered PreScission and Enterokinase cleavage sites, enabling controlled post-expression processing and generation of alternative protein forms derived from the same multitagged recombinant protein. As a proof of concept, p2GUS was evaluated using a truncated mitochondrial transcription factor A [mTFAM(p.1_41del)]. Recombinant protein production, purification, protease-mediated processing, and DNA-binding activity were assessed using multitagged mTFAM(p.1_41del) as a model substrate. Purification outcomes were compared with those obtained using the parental construct Precursor 1, encoding a single-His_10_–mTFAM(p.1_41del). Under the experimental conditions examined, the p2GUS-derived construct, purified using a simple single-microcentrifuge tube Ni–NTA batch procedure, showed improved recovery and yielded a processed His_10_–EK–mTFAM(p.1_41del) of substantially higher purity than the corresponding His_10_–mTFAM(p.1_41del) produced by Precursor 1. The purified recombinant protein retained DNA-binding activity, supporting preservation of its functional properties following expression, purification and storage. These results support proof-of-concept validation of p2GUS as a modular system for recombinant protein production, purification, and controlled post-expression processing. Although validated here using a DNA-binding protein, broader applicability remains to be established through evaluation with additional recombinant proteins.

**Graphical Abstract:**

**Figure:**
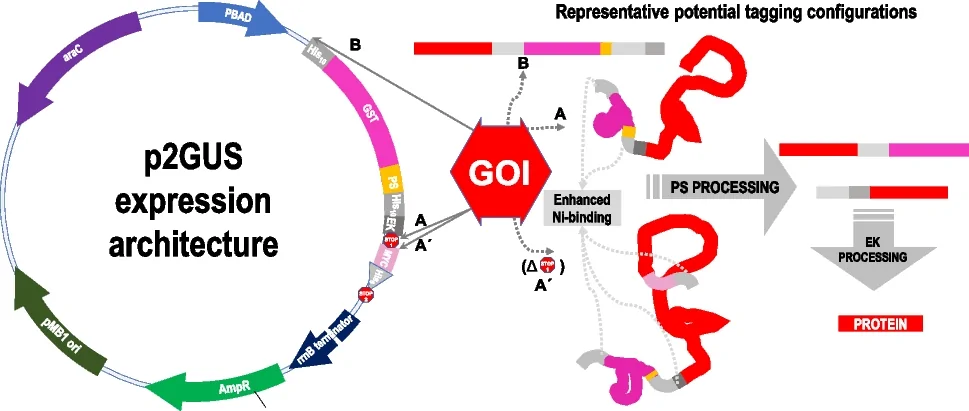

**Key points:**

• *Proof-of-concept validation of the modular p2GUS expression system*

• *Tandem His₁₀–GST–His₁₀ architecture improves Ni-NTA retention and purification*

• *Multitagged p2GUS-derived mTFAM retained DNA-binding activity*

**Supplementary Information:**

The online version contains supplementary material available at https://doi.org/10.1007/s00253-026-13968-x.

## Introduction

The production of functional recombinant proteins for understanding gene regulation, characterizing gene products, and elucidating protein structure–function relationships remains a major objective in both basic research and biotechnology. Prokaryotic and eukaryotic expression systems have been developed to address diverse research and industrial needs (Schütz et al. 2023; Khudainazarova et al. 2024), with prokaryotic hosts remaining widely used due to their simplicity, rapid growth (doubling times of approximately 20–60 min under optimal conditions), comparatively low production costs, and limited infrastructure requirements (Terpe 2006; Rosano and Ceccarelli 2014; Rosano et al. 2019; Ojima-Kato 2025).

Prokaryotic expression vectors typically exhibit a modular architecture comprising origins of replication, selectable markers, inducible promoters, cloning regions for insertion of the gene of interest (GOI), affinity and solubility tags, and protease cleavage sites (Guzman et al. 1995; Wang et al. 2009). Traditional restriction- and recombination-based cloning methods are increasingly being complemented or replaced by seamless and ligation-independent cloning technologies (Aslanidis and de Jong 1990; Lu 2005; Ferro-Gallego et al. 2024). Affinity and solubility tags facilitate purification, improve protein solubility and stability, and enable alternative purification and detection strategies (Kimple et al. 2013; Amarasinghe and Jin 2015; Raducanu et al. 2021), whereas site-specific protease cleavage sites permit controlled removal of affinity tags and generation of alternative protein variants (Raran-Kurussi and Waugh 2016). Together, these advances have facilitated the development of increasingly sophisticated expression systems that combine cloning flexibility, affinity-based purification, and controlled post-expression processing of recombinant proteins (Sørensen and Mortensen 2005; Smolke 2009).

Despite these advances, efficient production and purification of eukaryotic proteins containing multiple interaction domains remains challenging. Polyhistidine-based purification remains attractive because of its simplicity, speed, scalability, and low cost (Crowe et al. 1994). However, single His-tag configurations may exhibit reduced retention under highly stringent purification conditions and can co-purify endogenous host proteins with intrinsic metal-binding properties (Leonhardt et al. 2023). Such limitations are particularly relevant for proteins containing multiple protein- and nucleic acid-binding domains, where contaminating proteins may interfere with downstream biochemical analyses. Consequently, there remains interest in modular expression systems that combine precise cloning strategies, regulated expression, and alternative affinity-tag architectures designed to improve protein solubility and purification performance while retaining post-expression processing flexibility.

Here, p2GUS, a modular expression system generated through targeted modification of Precursor 1, a parental pBAD-derived vector, is described. The principal design feature incorporated into p2GUS is a tandem His₁₀–GST–His₁₀ affinity architecture developed to enhance interaction with Ni–NTA matrices while allowing flexible downstream processing. Unlike conventional single-tag configurations, this arrangement was designed to facilitate more stringent purification conditions through the incorporation of two polyhistidine modules flanking an intervening GST domain. The GST module serves primarily as a spacer separating the two His₁₀ tags, while also providing solubility-enhancing properties and compatibility with post-expression processing strategies. Although both components contribute to the overall architecture, the central feature of p2GUS is the tandem His₁₀ organization, which was specifically designed to increase the effective histidine content available for interaction with immobilized metal affinity matrices**.** Engineered protease cleavage sites and modular insertion positions were incorporated into the overall vector architecture to facilitate controlled post-expression processing of recombinant proteins and recovery of alternative protein forms from a common recombinant precursor (Raran-Kurussi and Waugh 2016).

The system retains the arabinose-inducible pBAD promoter (Guzman et al. 1995), enabling regulated recombinant protein production in *Escherichia coli*. Its modular architecture supports potential positional insertion of coding sequences within the expression cassette through seamless cloning strategies, providing flexibility in construct design and tag arrangement. Together, these features provide flexibility for recombinant protein production and post-expression processing through site-specific protease cleavage. Given the diversity of recombinant proteins and their expression requirements, comprehensive validation across multiple protein classes falls beyond the scope of a single study. Therefore, the present work was designed as a proof-of-concept evaluation of the p2GUS system using a truncated mitochondrial transcription factor A [mTFAM(p.1_41del)] as a representative and experimentally challenging DNA-binding protein. Recombinant protein production, purification performance, functional validation, and post-purification processing were assessed using this construct as a model substrate. Accordingly, the broader applicability of the system remains to be established through evaluation with additional recombinant proteins.

## Materials and methods

### Primers, plasmids, special reagents, and bacterial strains

All primers are listed in Supplementary Table S1, plasmids in Table 1 and Supplementary Table S2, and special reagents in Supplementary Table S3. *Escherichia coli* DH5α was used for plasmid propagation and chemical transformation, whereas *Escherichia coli* BL21 (DE3) was used for recombinant protein expression.

**Table 1:** Relevant plasmids used in this study

Plasmid name	Relevant use
Precursor 1: p2GUS-pr1-His10-*mTfam*(c.1_123del)*	Reference expression construct producing His_10_-mTFAM(p.1_41del)
p2GUS: p2GUS-His10-GST-PS-His10-EK-*mTfam*(c.1_123del)	Modular expression system constructed and evaluated in this study

*This plasmid was kindly provided by Dr. Vila-Sanjurjo (see Acknowledgements)

This table includes data previously published in Zenodo (https://doi.org/10.5281/zenodo.19160729)

### *Construction of p2GUS-His*_*10*_*-GST-PS-His*_*10*_*-EK-mTfam(c.1_123del)*

**Fig. 1:**
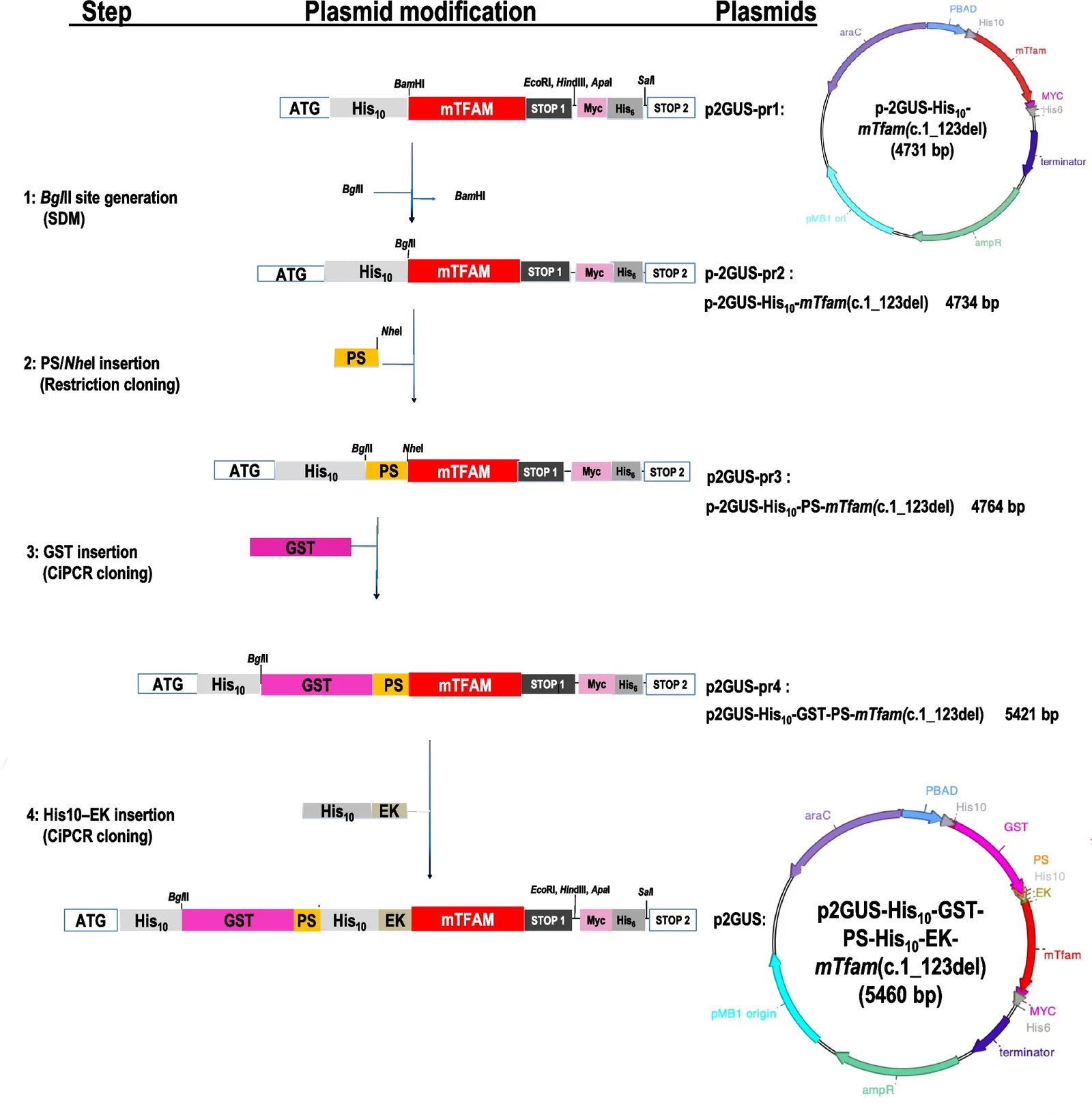
Stepwise construction scheme of p2GUS-His_10_-GST-PS-His_10_-EK-m*Tfam*(c.1_123del) (left, cloning strategy; center, genetic modification introduced at each step; right, resulting plasmids Step 1 generation of p2GUS-pr2-His_10_-m*Tfam(*c.1_123del) by *Bam*HI → *Bgl*II substitution using SDM and primers SDM-*Bgl*II. Step 2 generation of p2GUS-pr3-His_10_-PS-m*Tfam*(c.1_123del) by *Bgl*II/*Pvu*II restriction cloning of a PCR-derived PS/*Nh*eI fragment amplified with primers RE-PS/*Nhe*I. Step 3 generation of p2GUS-pr4-His_10_-GST-PS-m*Tfam*(c.1_123del) by insertion of a GST fragment amplified from pGEX-6P-1 using CiPCR and primers CiPCR-GST. Step 4 generation of p2GUS-His_10_-GST-PS-His_10_-EK-m*Tfam*(c.1_123del) by insertion of a His_10_-EK fragment using CiPCR and primers CiPCR-His_10_-EK. Transformation products from steps 1 to 4 were propagated in *Escherichia coli* DH5α. PS, PreScission protease cleavage site; GST, glutathione S-transferase; EK, enterokinase cleavage site; SDM, site-directed mutagenesis; CiPCR, circular PCR

The p2GUS expression architecture was established through sequential modification of the Precursor 1 construct, p2GUS-pr1 (p2GUS-His_10_-m*Tfam*(c.1_123del)), using site-directed mutagenesis, restriction/ligation cloning, and Circular PCR (CiPCR)-mediated assembly (Fig. 1). Detailed cloning procedures, intermediate constructs, screening strategies, and sequence verification data are provided in the Supplementary File.

Sequences and annotations of the plasmid constructs generated in this study have been deposited in Zenodo (https://doi.org/10.5281/zenodo.19160729). Information regarding the availability of physical plasmids is provided in the Data Availability section.

### Induction and expression of r-mTFAM

*Escherichia coli* BL21 (DE3) cells harboring p2GUS-His_10_-GST-PS-His_10_-EK-*mTfam*(c.1_123del) or Precursor 1 were grown and induced by arabinose.

### *Expression and purification of r-His*_*10*_*-mTFAM(p.1_41del) from Precursor 1 plasmid*

Cultures (100 mL for time-course studies, 500 mL for large-scale) in LB containing 100 µg/mL ampicillin were incubated at 37 °C and 200 rpm. Induction was performed at OD_600_ = 0.4 with 0.2% (w/v) arabinose. Samples were collected hourly (time-course) and lysed in 20 mM Tris–HCl pH 7.9, 0.5 M NaCl, 100 mM KCl, 1 µg/mL leupeptin, 1 µg/mL aprotinin, 1 mM PMSF, normalized according to the OD_600_ value of the time-zero sample. Lysis was performed by sonication (50% amplitude, 4 × 4 s pulses, with 5 s cooling intervals), 3 freeze–thaw cycles, and centrifugation at 15,000 × g 20 min at 4 °C. Supernatants were mixed with SDS-PAGE buffer, denatured 95 °C 2 min; and analyzed in 12% (w/v) SDS-PAGE/Western blot using anti-polyhistidine-HRP antibody (1:5000, Supplementary Table S3).

For large-scale expression and purification, 500 mL preparative cultures were harvested 3 h post-induction and lysed in 10 mL lysis buffer by sonication, freeze–thaw, followed by centrifugation. Ni–NTA purification was performed using FPLC or gravity columns with 200–500 µL of resin. Washes were performed sequentially, first with lysis buffer supplemented with 10% (v/v) glycerol, followed by additional washes with lysis buffer supplemented with 10 mM imidazole. FPLC elution was performed with 10–500 mM imidazole gradient, whereas gravity-flow elution used lysis buffer containing 0.5 M imidazole. Fractions were analyzed by SDS-PAGE together with concentrated (YM10, Supplementary Table S3) and desalted (PD-10, Supplementary Table S3) samples.

### *Expression and purification of multitagged r-His*_*10*_*-GST-PS-His*_*10*_*-EK- and single-tagged r-His*_*10*_*-EK-mTFAM(p.1_41del) from p2GUS-His*_*10*_*-GST-PS-His*_*10*_*-EK-mTfam(c.1_123del)*

Cultures (100 mL) were induced with 0.2% (w/v) arabinose and sampled hourly for 8 h for Western blot analysis using anti-GST antibody (1:2500 dilution, Supplementary Table S3). For this analysis, cells were lysed using a lysis buffer composed of 50 mM Tris–HCl pH 7.9, 0.5 M NaCl, 1 µg/mL leupeptin, 1 mM PMSF, and 1 µg/mL aprotinin. Cell pellets were resuspended in lysis buffer, normalized to the OD_600_ of the time-zero sample, and disrupted by sonication, freeze–thaw cycles, and centrifugation.

For large-scale expression and purification, 500 mL preparative cultures were induced 2 h, harvested, and resuspended in 10 mL lysis buffer described above. Cells were lysed by sonication, followed by freeze–thaw cycles and centrifugation to obtain clarified supernatants.

The supernatant was incubated with 200 µL of Ni–NTA beads for 2 h at 4 °C under slow rotation. After gentle centrifugation, the supernatant was removed and stored for later analysis. The beads were transferred to a 1.5-mL microcentrifuge tube and washed twice with 1.3 mL of lysis buffer containing 1% (v/v) Triton X-100, twice with 1.3 mL of lysis buffer alone, and twice with 1.3 mL of lysis buffer containing 50 mM imidazole. After each wash, the beads were gently centrifuged and the supernatants were discarded. Elution was performed twice in the same microcentrifuge tube using 200 µL of 1 M imidazole. The eluates were pooled and analyzed by SDS-PAGE.

Eluates were subsequently incubated with 100 µL of glutathione-agarose pre-equilibrated in PBS in a 1.5-mL microcentrifuge tube for 2 h at 4 °C under gentle agitation. The beads were washed twice with lysis buffer containing 1% (v/v) Triton X-100 and twice with PreScission protease cleavage buffer. Proteolytic cleavage was carried out at 4 °C by incubating the glutathione-agarose-bound protein with 70 µL of PreScission protease cleavage buffer and 5 µL of PreScission protease (Supplementary Table S3) for 24 h under gentle rotary agitation in the same microcentrifuge tube. Following this first incubation, the eluate was collected, and the beads were subsequently incubated with an additional 70 µL of cleavage buffer for a further 12 h without the addition of fresh protease, taking advantage of the GST-tagged protease remaining bound to the beads. After collection of the second eluate, both fractions were supplemented with 25% (v/v) glycerol and stored at −80 °C. Protein expression and purity were analyzed and confirmed by SDS-PAGE.

### *Electrophoretic mobility shift assays (EMSA) with r-His*_*10*_*-GST-PS-His*_*10*_*-EK-mTFAM(p.1_41del)*

Double-stranded DNA fragments (191 bp) generated by PCR and treated with ExoSAP-IT (1 h at 37 °C) or a pool of small DNA fragments (100–600 bp) obtained by sonication of genomic DNA extracted from *Escherichia coli* DH5α, were used in the assays. DNA concentrations were estimated by agarose gel electrophoresis and adjusted to the desired working concentrations.

Increasing concentrations of r-His_10_-GST-PS-His_10_-EK-mTFAM(p.1_41del) protein, purified as described above, were incubated with the DNA probes in binding buffer containing 1× PCR Buffer 2 (New England Biolabs), 1 mM DTT, 5% (v/v) glycerol, 0.05 mg/mL BSA, and EDTA-free protease inhibitor cocktail according to the manufacturer's recommendations (Supplementary Table S3). Binding reactions (20 µL final volume) were assembled using 200 ng of the PCR-generated DNA fragment or approximately 300 ng of the sonicated *E. coli* genomic DNA pool together with increasing concentrations of r- His_10_-GST-PS-His_10_-EK-mTFAM(p.1_41del) (approximately 0.12, 0.25, 0.5, and 1 µM). Reactions were incubated at room temperature for 60 min to allow protein–DNA complex formation.

Given the non-sequence-specific DNA-binding properties of mTFAM, no defined target sequence was required for probe design. Instead, DNA binding was evaluated as a function of protein concentration using the uncleaved r-His_10_-GST-PS-His_10_-EK-mTFAM(p.1_41del) fusion protein. Control reactions included DNA probes without protein to assess probe mobility, r-His_10_-GST-PS-His_10_-EK-mTFAM(p.1_41del) protein alone, and heat-denatured r-His_10_-GST-PS-His_10_-EK-mTFAM(p.1_41del) incubated with the corresponding DNA probes.

Prior to loading, 1 µL of glycerol was added to each reaction, and the entire sample was loaded onto a native 1.4% (w/v) agarose gel prepared in 0.5× TBE buffer. Electrophoresis was performed at a constant voltage of 20 V while maintaining the gel at ≤0 °C to preserve protein–DNA complex stability. Gels were stained with RedSafe and visualized under UV illumination.

### *EMSA-pull-down hybrid assay with r-His*_*10*_*-GST-PS-His*_*10*_*-EK-mTFAM(p.1_41del)*

To evaluate the ability of r-His_10_-GST-PS-His_10_-EK-mTFAM(p.1_41del) to bind DNA while retaining Ni–NTA-binding capability, a pull-down/EMSA hybrid assay was performed. Binding reactions were assembled using approximately 300 ng of a pool of 100–300 bp double-stranded DNA fragments obtained from *Escherichia coli* DH5α genomic DNA and approximately 2 µg of r-His_10_-GST-PS-His_10_-EK-mTFAM(p.1_41del) immobilized on Ni–NTA resin following purification from p2GUS. Reactions were carried out in binding buffer containing 1× NEBuffer 2 (New England Biolabs), 1 mM DTT, 0.1 mg/mL BSA, 5% (v/v) glycerol, and EDTA-free protease inhibitor cocktail according to the manufacturer’s recommendations (Supplementary Table S3). Samples were incubated at room temperature for 90 min to allow protein–DNA complex formation. The resin was subsequently recovered by centrifugation, and the supernatant fraction was collected for analysis. The resin was then washed with binding buffer to remove residual unbound DNA. Samples corresponding to the input DNA, the collected supernatant fraction, and r- His_10_-GST-PS-His_10_-EK-mTFAM(p.1_41del) –DNA complexes retained on the Ni–NTA matrix were analyzed by native agarose gel electrophoresis (1.6% w/v agarose in 0.5× TBE buffer). DNA was visualized by RedSafe staining under UV illumination. Control reactions lacking recombinant protein were processed in parallel to evaluate non-specific DNA retention by the Ni–NTA matrix. Recovery of DNA in the resin-bound fraction was used as an indicator of protein-mediated DNA capture.

## Results

### *Construction of the p2GUS-His*_*10*_*-GST-PS-His*_*10*_*-EK-mTfam(c.1_123del) multitagged expression system*

**Fig. 2:**
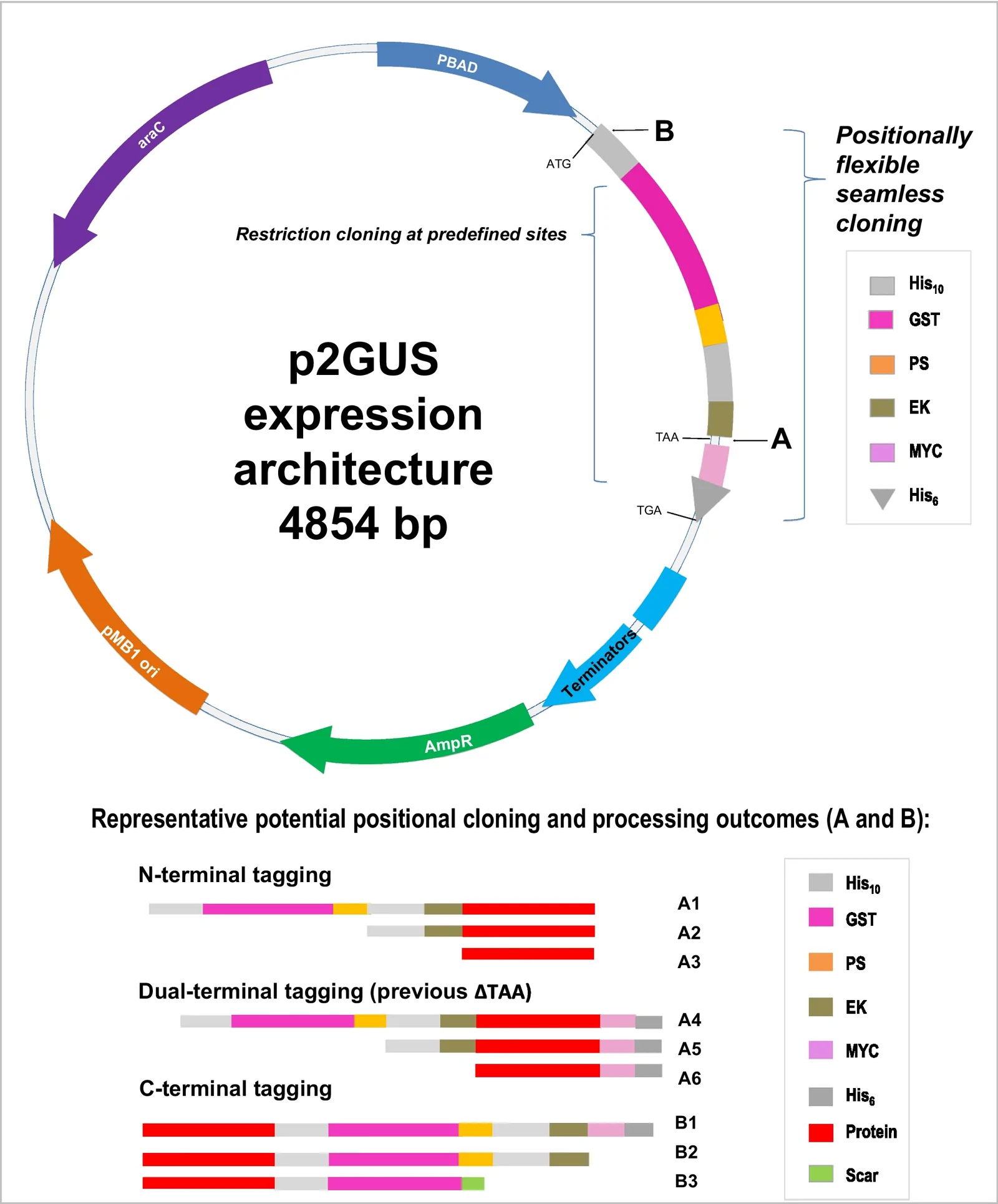
Modular architecture and potential cloning flexibility of p2GUS. Top, schematic representation of the p2GUS expression architecture. Bottom, representative positional cloning and potential processing outcomes generated by insertion of genes of interest at positions A and B. Alternative N-terminal, C-terminal, and dual-terminal tagging configurations are shown. PS, PreScission protease cleavage site; EK, enterokinase cleavage site; GOI, gene of interest

Sequential modification of the reference construct Precursor 1 (p2GUS-pr1-His_10_-m*Tfam*(c.1_123del)) generated the p2GUS expression architecture characterized in this study (Figs. 1 and 2 and Table 1). The resulting system was designed with a modular architecture that enables incorporation of genes of interest into the expression cassette for production of tagged recombinant proteins. Furthermore, for each particular construct, it facilitates recombinant protein purification and controlled post-expression processing of the encoded multitagged recombinant protein, enabling recovery of distinct protein forms from the same expression batch.

Construction was achieved through a combination of site-directed mutagenesis, restriction/ligation cloning, and CiPCR-mediated assembly, yielding a series of intermediate precursor constructs prior to generation of the final p2GUS-His_10_-GST-PS-His_10_-EK-*mTfam*(c.1_123del) expression construct (Fig. 1). Detailed construction procedures and validation data are provided in the Supplementary File (Supplementary Methods, Supplementary Results, and Supplementary Fig. S1).

### Cloning strategies supported by the p2GUS system

The modular architecture of p2GUS supports both seamless and restriction enzyme-based cloning strategies (Fig. 2). Coding sequences could potentially be incorporated into different locations within the expression cassette, providing flexibility in construct design and tag arrangement depending on the cloning strategy employed. Engineered protease cleavage sites further permit controlled post-expression conversion of multitagged recombinant proteins into alternative protein forms. Whereas restriction enzyme-based cloning is limited to predefined insertion sites (*Bgl*II, *Eco*RI, *Hind*III, *Apa*I and *Sal*I), seamless assembly approaches are not constrained by specific insertion sites.

### Production of recombinant single- and multitagged mTFAM proteins

**Fig. 3:**
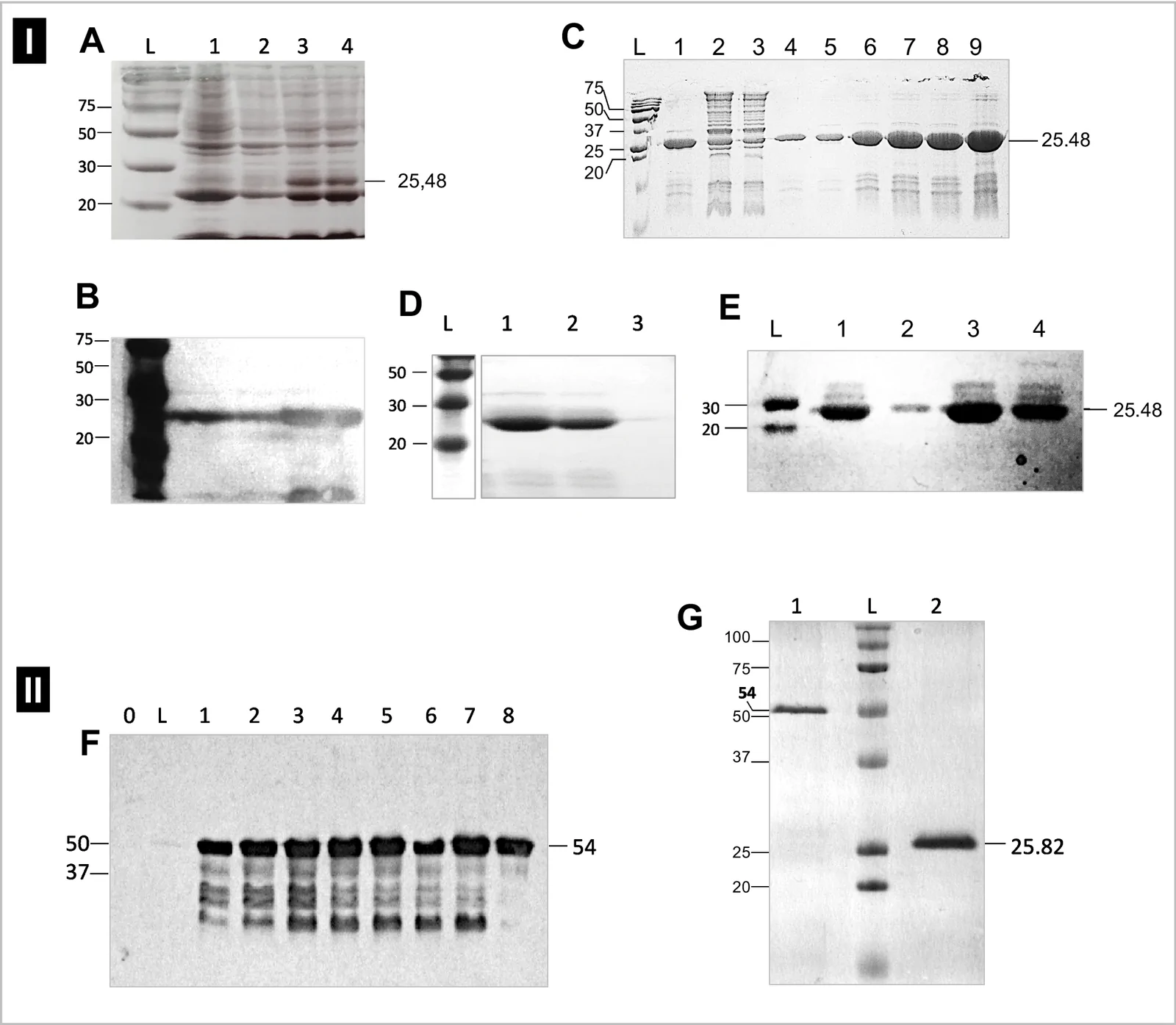
Expression, purification, and processing of recombinant mTFAM(p.1_41del) derivatives.** I** Expression and purification of r-His_10_-mTFAM(p.1_41del) encoded by the single-tagged construct Precursor 1 **A** SDS-PAGE analysis of arabinose-induced expression of r-His_10_-mTFAM(p.1_41del) in *Escherichia coli* BL21 harvested after 1–4 h induction (lanes 1–4). **B** Western blot corresponding to panel A probed with anti-His-HRP. **C** SDS-PAGE analysis of Ni–NTA gravity-flow and FPLC fractions (lane 1, gravity-flow eluate; lanes 2–3, gravity-flow washes; lanes 4–9, FPLC fractions). **D** SDS-PAGE analysis of Ni–NTA re-purification fractions (lane 1, pooled fractions; lane 2, 500 mM imidazole eluate; lane 3, 10 mM imidazole wash). **E** SDS-PAGE analysis of Amicon-concentrated samples (lanes 1 and 3, pooled fractions; lane 2, flow-through; lane 4, desalted fraction). **II** Expression, purification, and processing of r-His_10_-GST-PS-His_10_-EK-mTFAM(p.1_41del) produced from p2GUS-His_10_-GST-PS-His_10_-EK-m*Tfam*(c.1_123del). **F** Western blot analysis of 0–8 h induction of r-His_10_-GST-PS-His_10_-EK-mTFAM(p.1_41del) in *Escherichia coli* BL21 probed with anti-GST. **G** Recombinant proteins obtained from large-scale cultures (lane 1, Ni–NTA-purified multitagged r-His_10_-GST-PS-His_10_-EK-mTFAM(p.1_41del); lane 2, r-His_10_-EK-mTFAM(p.1_41del) obtained following GST-bead binding and PreScission protease cleavage. L, molecular-weight marker; Gt, glutathione. Uncropped SDS-PAGE gels and Western blots are provided in the Supplementary File

Recombinant N-terminally His₁₀-mTFAM(p.1_41del) (r-His₁₀-mTFAM(p.1_41del)) was initially produced using Precursor 1 (p2GUS-pr1-His₁₀-*mTfam*(c.1_123del)) (Fig. 3I). The p2GUS construct, p2GUS-His_10_-GST-PS-His_10_-EK-*mTfam*(c.1_123del), enabled production of both a multitagged protein, r-His₁₀-GST-PS-His₁₀-EK-mTFAM(p.1_41del), and a processed single-tagged derivative, r-His₁₀-EK-mTFAM(p.1_41del), from the same expression batch (Fig. 3II).

### Expression and purification of r-His₁₀-mTFAM from Precursor 1

Time-course induction experiments identified 3 h of arabinose induction as optimal for expression of His₁₀-mTFAM(p.1_41del) (Fig. 3I(A) and I(B)). Purification using Ni–NTA affinity chromatography in gravity-flow and FPLC formats yielded the expected 25.48 kDa recombinant protein, although additional protein species remained detectable following purification (Fig. 3I(C)). Additional Ni–NTA repurification, desalting, and concentration steps reduced low-molecular-weight contaminants but did not completely eliminate higher-molecular-weight species (Fig. 3I(D) and 3I(E)). Depending on the purification strategy employed, protein yields ranged from approximately 0.5–4 mg of r-His₁₀-mTFAM(p.1_41del)/L of culture, with purities between 65 and 85%. Considerable protein losses were observed during downstream processing, particularly during fraction selection and sample handling steps.

### *Expression and purification of recombinant proteins from p2GUS-His*_*10*_*-GST-PS-His*_*10*_*-EK-mTfam(c.1_123del)*

In contrast, expression of p2GUS-His_10_-GST-PS-His_10_-EK-*mTfam*(c.1_123del) products yielded the multitagged recombinant protein r-His₁₀-GST-PS-His₁₀-EK-mTFAM(p.1_41del), which migrated at approximately 54 kDa and was purified by Ni–NTA batch binding using a simple microtube-based workflow. Stringent washing conditions produced preparations of approximately 85–90% purity with yields of 8–12 mg/L of culture (Fig. 3II(G), lane 1). Following PreScission protease cleavage, the processed derivative r-His₁₀-EK-mTFAM(p.1_41del) (25.8 kDa) was recovered at near-homogeneous purity (>99%) and approximately 90% yield relative to the multitagged precursor (~5-mg protein/L of culture; Fig. 3II(G), lane 2). Together, these results demonstrate the ability of p2GUS to generate both highly enriched precursor proteins and highly purified processed derivatives within a single workflow.

### Functional characterization of recombinant mTFAM reveals concentration-dependent DNA binding

**Fig. 4:**
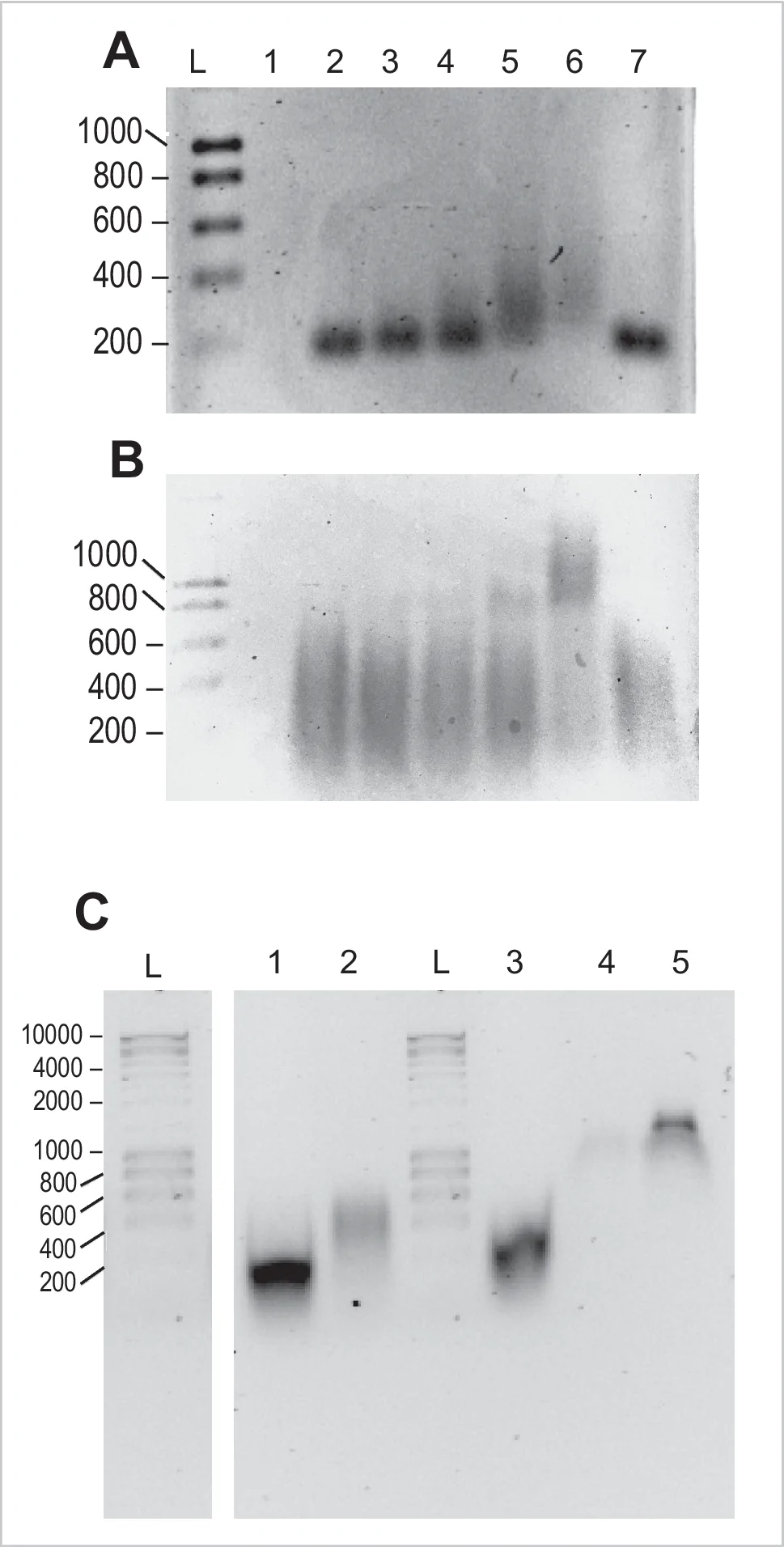
Functional characterization of recombinant mTFAM derivatives produced using p2GUS-His_10_-GST-PS-His_10_-EK-m*Tfam*(c.1_123del).** A** Electrophoretic mobility shift assay (EMSA) performed with r-His_10_-GST-PS-His_10_-EK-mTFAM(p.1_41del) and a 191-bp PCR-derived double-stranded DNA fragment and analyzed by 1.4% agarose gel (lane 1, 1 µM r-His_10_-GST-PS-His_10_-EK-mTFAM(p.1_41del); lane 2, 200 ng DNA; lanes 3, 4, 5 and 6, 0.12, 0.25, 0.5, and 1 µM r-His_10_-GST-PS-His1_0_-EK-mTFAM(p.1_41del), respectively, each incubated with 200 ng DNA; lane 7, 1 µM heat-denatured r-His_10_-GST-PS-His_10_-EK-mTFAM(p.1_41del) incubated with 200 ng DNA. **B** EMSA performed under the same conditions as in panel A using fragmented *Escherichia coli* genomic DNA (approximately 100–600 bp; 250 ng). **C** EMSA–pull-down hybrid assay using approximately 2 µg of r-His_10_-GST-PS-His_10_-EK-mTFAM(p.1_41del) immobilized on Ni–NTA resin and fragmented *Escherichia coli* genomic DNA (approximately 100–300 bp; 300 ng) (lane 1, supernatant from the control reaction lacking recombinant protein; lane 2, supernatant from the reaction containing r-His_10_-GST-PS-His_10_-EK-mTFAM(p.1_41del); lane 3, DNA alone; lane 4, DNA retained on Ni–NTA resin from the control reaction lacking recombinant protein; lane 5, DNA retained on Ni–NTA resin from the reaction containing r-His_10_-GST-PS-His_10_-EK-mTFAM(p.1_41del)). L, DNA ladder. DNA ladder sizes are indicated in bp. Uncropped agarose gel images are provided in the Supplementary File

The DNA-binding activity of r-His₁₀-GST-PS-His₁₀-EK-mTFAM(p.1_41del) was assessed by electrophoretic mobility shift assay (EMSA) using a 191 bp PCR-derived DNA fragment. Very low DNA mobility was observed at low protein concentrations (<0.25 µM) (Fig. 4A, lane 3). As r-His₁₀-GST-PS-His₁₀-EK-mTFAM(p.1_41del) concentration increased (0.12–1 µM), progressive retardation of DNA migration became evident (Fig. 4A, lanes 3–6). The same behavior was observed for an analogous EMSA using a pool (100–600 bp) of *Escherichia coli* genomic DNA (Fig. 4B). Rather than producing a discrete shifted complex, r-His₁₀-GST-PS-His₁₀-EK-mTFAM(p.1_41del) generated a gradual reduction in electrophoretic mobility accompanied by increasing signal dispersion at higher protein concentrations. This behaviour is consistent with the ability of TFAM to bind non-sequence-specific DNA, induce pronounced DNA bending, and promote higher-order DNA compaction through cooperative interactions (Ngo et al. 2014; Choi and Garcia-Diaz 2022; Kozhukhar and Alexeyev 2023). Control reactions lacking or containing heat-denatured r-His₁₀-GST-PS-His₁₀-EK-mTFAM(p.1_41del) (Fig. 4A and B, lanes 2 and 7, respectively) displayed a single band corresponding to free DNA, confirming that the observed mobility changes depended on the presence of functionally active recombinant protein.

These results indicate that r-His₁₀-GST-PS-His₁₀-EK-mTFAM(p.1_41del) produced and purified using p2GUS-His_10_-GST-PS-His_10_-EK-*mTfam*(c.1_123del) retained DNA-binding activity following expression, purification, and storage.

### EMSA–pull-down hybrid assay reveals stable TFAM–DNA association

To further evaluate mTFAM functionality, an EMSA–pull-down hybrid assay was performed using r-His₁₀-GST-PS-His₁₀-EK-mTFAM(p.1_41del) immobilized on Ni–NTA resin. R-His₁₀-GST-PS-His₁₀-EK-mTFAM(p.1_41del)-bound resin was incubated for 1 h with a heterogeneous mixture of 100–400 bp *Escherichia coli* genomic DNA fragments. Following incubation, the supernatant containing unbound material was collected, and the resin was washed twice with binding buffer prior to electrophoretic analysis.

Analysis of the resin-associated fraction revealed a strong DNA signal, indicating retention of DNA through interaction with immobilized r-His₁₀-GST-PS-His₁₀-EK-mTFAM(p.1_41del) (Fig. 4(C)). As a negative control, Ni–NTA resin subjected to the same procedure in the absence of recombinant TFAM showed no detectable DNA retention, confirming that DNA capture was dependent on the presence of TFAM.

The supernatant fraction retained detectable DNA, although at reduced intensity relative to the input sample, consistent with partial retention of DNA by resin-bound r-His₁₀-GST-PS-His₁₀-EK-mTFAM(p.1_41del). Notably, DNA present in the supernatant from r-His₁₀-GST-PS-His₁₀-EK-mTFAM(p.1_41del)-containing samples displayed reduced electrophoretic mobility relative to free DNA, suggesting the presence of soluble mTFAM–DNA complexes released during handling that remained stable throughout electrophoresis.

Together with the EMSA results, these observations support the DNA-binding competence of r-His₁₀-GST-PS-His₁₀-EK-mTFAM(p.1_41del) produced using p2GUS-His_10_-GST-PS-His_10_-EK-*mTfam*(c.1_123del) and indicate that r-His₁₀-GST-PS-His₁₀-EK-mTFAM(p.1_41del) retained DNA-binding activity both in solution and while immobilized on the affinity matrix. Notably, DNA-binding activity was retained after storage of the immobilized r-His₁₀-GST-PS-His₁₀-EK-mTFAM(p.1_41del) protein on Ni–NTA resin at 4 °C for at least 2 weeks, whereas soluble r-His₁₀-GST-PS-His₁₀-EK-mTFAM(p.1_41del) DNA-binding activity after storage at −80 °C for at least 1 month.

## Discussion

The recombinant production of highly purified and functional eukaryotic DNA-binding proteins in bacterial hosts frequently remains challenging despite the availability of numerous expression systems. In the present study, several conventional vector configurations derived from the pBAD, pET, and pGEX families were evaluated using mouse mitochondrial transcription factor A (mTFAM) as a representative DNA-binding protein. Although recombinant expression was achieved in all cases, purification outcomes differed among the tested constructs. The best performance was obtained with a pBAD-derived vector encoding a single His₁₀ tag (Fig. 3I). Nevertheless, this configuration did not provide the combination of product enrichment, recovery, and workflow simplicity required for the intended applications. This prompted the development of an alternative expression and purification strategy (Fig. 3).

These observations are consistent with previous reports describing the difficulties associated with recombinant production of eukaryotic proteins in bacterial hosts (Makrides 1996; Sørensen and Mortensen 2005; Rosano and Ceccarelli 2014). Such difficulties may be particularly relevant for DNA-binding proteins, whose intrinsic interactions with nucleic acids can complicate purification procedures and downstream biochemical analyses. Such properties can reduce the effectiveness of conventional affinity purification strategies, even when strong expression systems and widely used affinity tags are employed. Previous studies have also noted that purification of DNA-binding proteins frequently relies on multistep workflows that may result in substantial cumulative protein losses (Gadgil et al. 2001). In this context, the development of strategies capable of simplifying purification while maintaining protein purity and functionality remains of considerable interest.

The comparative evaluation of these expression systems highlighted useful features within each platform. The pGEX system provides a GST fusion module that may enhance protein solubility and enables efficient tag removal through a PreScission protease cleavage site (Smith and Johnson 1988). pET-derived vectors enable robust expression and straightforward immobilized metal affinity purification (Studier and Moffatt 1986), whereas pBAD-derived systems offer tightly regulated arabinose-inducible expression (Casadaban and Cohen 1980; Guzman et al. 1995). The p2GUS construct was conceived as a proof-of-concept design integrating selected advantages of these established approaches within a single modular architecture (Fig. 2). Importantly, no individual construct evaluated in this study simultaneously combined inducible expression control, dual polyhistidine affinity modules, removable fusion elements, and positionally flexible seamless-cloning compatibility within the same vector framework.

A central feature of the p2GUS design is the incorporation of two His₁₀ tags separated by a GST-containing region (Fig. 2). The rationale for this architecture was based on studies showing that the performance of polyhistidine-mediated purification depends not only on tag composition but also on the number and spatial arrangement of metal-binding motifs. Single His₆ tags typically display affinities within the low micromolar range under monovalent binding conditions (Nieba et al. 1997; Dorn et al. 1998; Krishnan et al. 2007; Knecht et al. 2009). Extension to longer tags such as His₁₀ can improve binding and purification performance (Grisshammer and Tucker 1997; Krishnan et al. 2007). Importantly, the affinity enhancement associated with tandem polyhistidine architectures is influenced not only by tag multiplicity but also by the spatial arrangement of the metal-binding motifs. When multiple coordination sites are presented in a suitable configuration, avidity effects can reduce effective dissociation rates and increase apparent binding strength (Lata and Piehler 2005; Lata et al. 2005; Wasserberg et al. 2017). Tandem histidine-tag constructs have previously been reported using relatively short peptide spacers; for example, Khan et al. (2006) described a tandem His₆ architecture in which the two affinity modules were separated by an 11-amino-acid linker. In contrast, the p2GUS design incorporates a substantially larger intervening region consisting of a 231-amino-acid GST module positioned between two His₁₀ tags (Fig. 2). This organization was intended not only to increase the number of available histidine residues but also to maximize physical separation of the affinity motifs, potentially reducing steric constraints and improving accessibility of the histidine residues for interaction with the matrix, while simultaneously providing solubility-enhancing properties and compatibility with post-expression processing. Tandem polyhistidine constructs may therefore exhibit enhanced matrix retention compared with single-tag configurations (Khan et al. 2006). The experimental results obtained with p2GUS-His_10_-GST-PS-His_10_-EK-*mTfam*(c.1_123del) (Fig. 3) are consistent with this design rationale, although direct evaluation of spacer-length effects and quantitative characterization of matrix-binding avidity were beyond the scope of the present study.

Consistent with this design rationale, the Precursor 1 construct containing a single His₁₀ tag exhibited limited purification performance, yielding protein preparations of approximately 65–85% apparent purity and recoveries of approximately 0.5–4 mg/L of culture (Fig. 3). Additional losses were frequently incurred during fraction-selection steps and downstream processing procedures such as desalting, concentration, and buffer-exchange operations, resulting in cumulative reductions in final protein recovery (Fig. 3I).

In contrast, the p2GUS-His_10_-GST-PS-His_10_-EK-*mTfam*(c.1_123del)-derived construct displayed substantially improved purification performance, allowing stringent washing conditions while maintaining efficient recovery of recombinant protein. Following Ni–NTA purification, preparations typically reached approximately 85–90% apparent purity, with yields of approximately 8–12 mg/L of culture. Subsequent processing of the purified fusion protein, r-His₁₀-GST-PS-His₁₀-EK-mTFAM(p.1_41del), generated a highly purified r-His₁₀-EK-mTFAM(p.1_41del) derivative approaching electrophoretic homogeneity (~99% apparent purity), with approximately 90% recovery during processing (approximately yield of 5 mg/L of culture, Fig. 3II). Notably, this level of enrichment was achieved using a comparatively simple workflow based on batch binding and small-volume elution, minimizing the need for extensive downstream handling.

Although the present work did not directly determine matrix-binding constants or quantitatively evaluate avidity effects, the observed purification behavior is consistent with the expected performance of a multivalent affinity architecture. Importantly, the purity, recovery, and overall yield obtained generated functionally active recombinant mTFAM in quantities well beyond those required for the biochemical analyses reported here (Figs. 3, 4).

An important practical aspect of the system is the simplicity of the purification workflow. A major objective during vector design was not only to improve protein purity but also to develop a rapid and experimentally accessible purification strategy that could be implemented using standard laboratory equipment. In the configuration evaluated here, protein capture, washing, and elution were performed entirely within a single microcentrifuge tube using Ni–NTA resin, without requiring dedicated chromatographic instrumentation during the primary purification stage. Furthermore, elution could be performed in relatively small buffer volumes, frequently reducing or eliminating the need for additional concentration procedures. The entire purification and post-expression processing workflow were carried out under non-denaturing conditions, allowing recovery of recombinant protein while preserving the DNA-binding activity subsequently demonstrated by EMSA and EMSA–pull-down analyses (Fig. 4). These characteristics simplify handling and may be particularly advantageous for laboratories lacking access to specialized protein purification infrastructure or for research groups that require recombinant proteins as experimental tools rather than as a principal research focus. Rather than relying on increasingly complex downstream purification schemes, the p2GUS design seeks to improve purification performance at the construct-design stage through improved retention during affinity purification while maintaining protein functionality. In addition to improving primary purification performance, the simplified workflow may reduce cumulative protein losses associated with repeated downstream manipulation steps, including fraction selection, desalting, concentration, and buffer exchange (Fig. 3II).

The results further indicate that high levels of purity can be achieved through a staged purification strategy. While Ni–NTA purification alone yielded preparations displaying high apparent purity, subsequent processing of the fusion construct enabled additional removal of residual contaminants and generated preparations approaching electrophoretic homogeneity. Notably, this increase in purity was achieved while maintaining approximately 90% recovery during conversion of the fusion protein into the processed derivative (Fig. 3II). In addition, the incorporation of protease cleavage sites provides flexibility by allowing the highly purified fusion protein to be converted into a less extensively tagged derivative after purification. Thus, affinity modules used during protein production can be selectively removed while retaining the purification advantages they provide during earlier workflow stages (Fig. 3II).

Beyond the specific mTFAM construct examined here, p2GUS was designed as a modular expression architecture. As illustrated in Fig. 2, genes of interest can potentially be introduced at alternative positions within the expression cassette through seamless cloning approaches. Consistent with this design flexibility, replacement of *mTfam* with the gene encoding the DNA-binding protein hPCBP1 has previously been reported within the same vector framework, yielding functional recombinant protein (Ferro-Gallego et al. 2024). Although only one specific configuration was experimentally validated in the present study, this modular organization provides flexibility for the generation of alternative recombinant protein formats and may facilitate future adaptation of the system to different production requirements. Such flexibility may be particularly useful when different tagging arrangements or post-expression processing strategies are required. The configuration selected for validation was considered particularly attractive because it combines enhanced purification potential with protease-mediated processing capabilities and the possibility of generating minimally modified or scarless protein termini under suitable construct configurations.

A key objective of the present work was not only to obtain recombinant protein but also to evaluate whether functional activity could be preserved following purification. Recombinant mTFAM obtained using p2GUS retained DNA-binding activity in electrophoretic mobility shift assays employing both a defined PCR-derived DNA fragment and heterogeneous bacterial genomic DNA preparations. Furthermore, DNA retention was observed in the EMSA–pull-down hybrid assay (Fig. 4). The concordant results obtained using two complementary approaches and two distinct DNA substrates support the conclusion that the purified protein retained biologically relevant DNA-binding activity. Similar expression–purification–functional validation workflows have proven necessary for other recombinant DNA-binding proteins, where demonstration of DNA-binding activity constitutes a critical step in confirming preservation of biological function following purification (Twerdochlib et al. 2003; Moghaddas Sani et al. 2018). While comprehensive biophysical characterization (e.g., by circular dichroism or size-exclusion chromatography) was beyond the scope of the present study, the combined observation of high purity and retained DNA-binding activity was considered sufficient for proof-of-concept validation of the recombinant product (Fig. 4).

The EMSA–pull-down assay provided additional evidence that recombinant TFAM retained DNA-binding activity while immobilized on the affinity matrix. In addition to DNA retention on the Ni–NTA resin, DNA present in the supernatant fraction displayed reduced electrophoretic mobility relative to the free DNA control. The absence of a comparable shift in the negative control suggests the presence of soluble TFAM–DNA complexes in the supernatant (Fig. 4). Although the origin of these complexes was not investigated further, they may reflect the presence of active mTFAM released from the affinity matrix during the assay. Together, these findings support preservation of DNA-binding competence in recombinant TFAM produced using p2GUS (Fig. 4).

Notably, DNA-binding activity was retained after storage of the immobilized protein on Ni–NTA resin at 4 °C for at least 2 weeks. Similarly, soluble r-mTFAM retained DNA-binding activity following storage at −80 °C for at least 1 month, as demonstrated by EMSA analyses performed using both PCR-derived and genomic DNA substrates (Fig. 4). These observations further support the suitability of the purified recombinant protein for downstream biochemical applications.

The present study was not designed to establish the general applicability of p2GUS across diverse protein families. Rather, it provides proof-of-concept validation using mTFAM, a DNA-binding protein for which the conventional constructs evaluated in this work did not provide the combination of purity, recovery, and workflow simplicity sought for subsequent applications. Accordingly, the present results should not be interpreted as evidence that similar improvements will necessarily be obtained for all recombinant proteins. Likewise, although the purification results are consistent with the expected behavior of a multivalent affinity architecture, direct biophysical characterization of the underlying binding mechanism was beyond the scope of the study. Additional investigations involving a broader range of recombinant proteins will be required to define the scope, limitations, and mechanistic basis of the approach more fully.

Overall, the results presented here provide proof-of-concept validation of the p2GUS design. By combining inducible expression control, a dual-His₁₀ affinity architecture, removable fusion modules, modular cloning flexibility, and a simple purification workflow, p2GUS enabled the production of highly purified and functionally active r-mTFAM. The system supported generation of recombinant protein preparations approaching electrophoretic homogeneity (~99% apparent purity) while maintaining approximately 90% recovery during post-expression processing. These findings demonstrate that rational optimization of construct architecture can facilitate the production of highly purified and functionally active r-mTFAM while simplifying downstream purification.

## Supplementary Information

Below is the link to the electronic supplementary material.

ESM 1 (DOCX 122 MB)

## Data Availability

Plasmid sequences and annotations have been deposited in Zenodo under https://doi.org/10.5281/zenodo.19160729. Physical plasmids are available from the corresponding author upon reasonable request.
