# Supplementary material for: Proof-of-concept validation of p2GUS, a modular expression system for enhanced Ni-binding purification of functional recombinant proteins in Escherichia coli
Source: Appl Microbiol Biotechnol. 2026 Aug 8;110:334. doi: 10.1007/s00253-026-13968-x (PMC13737311; doi:10.1007/s00253-026-13968-x)
Supplement: Supplementary file 1 [file 253_2026_13968_MOESM1_ESM.docx]

**Supplementary Information**

**1. Supplementary Materials and Methods	2**

**1.1 Construction of p2GUS vector	2**

**1.2 Supplementary Tables	4**

# 2. Supplementary Results 6

**2.1 Construction of the p2GUS multi-tagged	6**

**expression system from Precursor 1**

**2.2 Supplementary Figures	8**

**3. Uncropped images of Fig 3	10**

**1. Supplementary Materials and Methods**

**1.1 Construction of p2GUS vector**

p2GUS was constructed from p2GUS-pr1-His_10_-*mTfam*(c.1_123del), hereafter referred to as Precursor 1, through four sequential steps. Precursor 1 contains a partial *mTfam*(c.1_123del) ORF, is inducible by arabinose, and expresses recombinant N-terminally His_10_-tagged mTFAM**(p.1_41del).**

**Step 1**

p2GUS-pr2-His_10_-*mTfam*(c.1_123del) was generated by replacing *Bam*HI (359–364) with *Bgl*II in Precursor 1 using SDM (QuikChange®, Supplementary Table S3). PCR (50 µl) contained 125 ng each primer (SDM-*Bgl*II, Supplementary Table S1), 2.5 U Pfu Turbo DNA polymerase, 69 ng Precursor 1 template, 5 µl 10X buffer, and 1 µl each dNTP. Cycling: 95°C 30 s; 12 cycles, 95°C 30 s, 55°C 1 min, 68°C 5 min. Products were treated with 2 U *Dpn*I (Supplementary Table S3) at 37°C 2 h. After transformation, plasmid prep, and restriction analysis with *Bgl*II (Table 3), the final sequencing confirmed precursor 2 construct p2GUS-pr2-His_10_-*mTfam*(c.1_123del).

**Step 2**

p2GUS-pr3-His_10_-PS-*mTfam*(c.1_123del) was generated by inserting a 301-bp DNA fragment containing the PreScission (PS) protease cleavage sequence and the *Nhe*I restriction site into precursor 2 using restriction/ligation. PCR amplification of the PS/*Nhe*I insert (80 µl) contained 16 µl 5x buffer, 0.2 µM each primer (RE-PS/*Nhe*I, Supplementary Table S1), 0.2 mM each dNTP, 200 ng p2GUS-pr2-His_10_-*mTfam*(c.1_123del) template, and 2 U Takara DNA polymerase (Supplementary Table S3). Cycling: 95°C 5 min; 30 cycles, 95°C 30 s, 60°C 30 s, 72°C 1 min; final 72°C 10 min. Products were purified, digested with *Bgl*II and *Pvu*II (Supplementary Table S3), ligated with 30 ng linear vector using T4 DNA ligase (Supplementary Table S3), transformed, and analyzed by colony-PCR (RE-PS/*Nhe*I, Supplementary Table S1) and restriction analysis with *Nhe*I (Supplementary Table S3). Final sequencing of a purified plasmid confirmed precursor 3 construct p2GUS-pr3-His_10_-PS-*mTfam*(c.1_123del).

**Step 3**

p2GUS-pr4-His_10_-GST-PS-*mTfam*(c.1_123del) was generated by inserting the glutathione-S-transferase (GST) coding sequence (680 bp) into precursor 3 via CiPCR (Ferro-Gallego et al. 2024). PCR to amplify GST (50 µl), contained 10 µl 5x buffer, 0.2 µM primers (CiPCR-GST, Supplementary Table S1), 0.2 mM each dNTP, 50 ng pGEX-6P-1 (Supplementary Table S2) template, and 1.25 U Takara DNA polymerase. Cycling, 94°C 30 s; 40 cycles, 98°C 10 s, 54°C 30 s, 72°C 45 s; final 72°C 10 min. CiPCR to insert GST (25 µl): 5 µl 5X buffer, 0.2 mM each dNTP, 250 ng GST insert, 100 ng p2GUS-pr3-His_10_-PS-*mTfam*(c.1_123del) template, and 1.25 U Takara DNA polymerase. Cycling: 5 cycles, 95°C 1 min, 81°C 30 s, 65°C 30 s, 72°C 6 min; 10 cycles, 98°C 20 s, 81°C 15 s, 75°C 15 s, 72°C 6 min; final 72°C 15 min. *Dpn*I digestion, precipitation, transformation, colony-PCR analysis, miniprep plasmid isolation and final sequencing confirmed precursor 4 construct p2GUS-pr4-His_10_-GST-PS-*mTfam*(c.1_123del).

**Step 4**

p2GUS-His_10_-GST-PS-His_10_-EK-*mTfam*(c.1_123del) was generated by inserting a second His_10_-tag and (EK) cleavage site via CiPCR. CiPCR (25 µl): 5 µl 5x buffer, 0.2 µM each primer (CiPCR-10H/EK, Supplementary Table S1), 0.2 mM each dNTP, 100 ng p2GUS-pr4-His_10_-GST-PS-*mTfam*(c.1_123del) template, and 1.25 U of Takara DNA polymerase. Cycling: 95ºC 5 min; 5 cycles 95°C 1 min, 70°C 7.5 min; 10 cycles 98°C 20 s, 70°C 15 s, 72°C 7 min; final 72°C 15 min. After *Dpn*I digestion, precipitation, transformation, colony-PCRs with primers CiPCR-10H/EK1 for preselection, and with primers CiPCR-10H/EK2 for screening (Supplementary Table S1), restriction analysis with *Nhe*I confirmed the presence of the insert. After miniprep plasmid extraction, sequencing corroborated the final construct p2GUS-His_10_-GST-PS-His_10_-EK-*mTfam*(c.1_123del).

**1.2 Supplementary Tables**

**Supplementary Table S1. Primers used in the experiments**

| **Primer / (Step)** | **Sequence (5´-3´)** |
| --- | --- |
| **SDM-*Bgl*II / (1)**  (Chimeric (*Bgl*II sites in bold)) | F: *ggcggc***agatct***ttttccagcatgggtag*  R: *ctggaaaa***agatct***gccgccatgatgatg* |
| **RE-PS/*Nhe*I / (2)**  (PS/*Nh*e underlined) | F: tttt**agatct**ctggaagttctgttccaggggccc**gctagc**ttttccagcatgggtagcta  R: catacccat**cagctg**acttggagttag |
| **CiPCR-GST / (3)**  (Chimeric) | F: *catcatcatggcggcagatct-*tcccctatactagg  R: *aaagctagcgggcccctggaacagaacttc* |
| **CiPCR-His_10_-EK / (4)**  (F, Chimeric: His_10_-EK insert underlined. R, non-chimeric) | F: *gaagttctgttccaggggccc****-***catcaccatcaccatcaccatcaccatcac-gatgacgatgacaag-*ttttccagcatgggtagctatcca*  R: *aaagctagcgggcccctggaacagaacttc* |
| **CiPCR-His_10_-EK-1 / (4)**  (For colony-PCR) | F: ctaggctggaagttctgttccaggggcccg  R: ttttgaattcctctttatacttgctcacagcttcttt |
| **CiPCR-His_10_-EK-2 / (4)**  (For colony-PCR) | F: gaccaaagccatgacaaaaacg  R: tcgacggcgctattcagatc |

Primers from Sigma-Aldrich are shown in 5´-3´ direction. Plain text font-regions in chimeric primers are to amplify the insert, italic font-regions are homologous to the CiPCR template (if there is no plain text the sequence is common between both templates).

**Supplementary Table S2. Plasmids used or generated in this study**

| **Plasmid name** | **Source/supplier** |
| --- | --- |
| p2GUS-pr1-His10-*mTfam*(c.1_123del) | * |
| p2GUS-pr2-His10-*mTfam*(c.1_123del) | This study |
| p2GUS-pr3-His10-PS-*mTfam*(c.1_123del) | This study |
| p2GUS-pr4-His10-GST-PS-*mTfam*(c.1_123del) | This study |
| p2GUS-His10-GST-PS-His10-EK-*mTfam*(c.1_123del) | This study |
| pGEX-6P-1 | GE Healthcare |

* This vector was kindly provided by Dr. Vila-Sanjurjo (see Acknowledgements)

This table includes data previously published in Zenodo (<https://doi.org/10.5281/zenodo.19160729>).

.

**Supplementary Table S3. Special reagents**

| Name in the text | Full name | Supplier |
| --- | --- | --- |
| *Bam*HI | *Bam*HI Restriction Endonuclease | Roche |
| *Bgl*II | *Bgl*II Restriction enzyme | New England Biolabs |
| Big Dye | Big Dye Terminator v3.1 Cycle Sequencing Kit | 4336917, Applied Biosystems |
| *Dpn*I | *Dpn*I Restriction Endonuclease | Thermo Fisher Scientific |
| QuikChange® | QuikChange® Site-Directed Mutagenesis Kit | Stratagene |
| GST antibody | GST (B14): sc-138 | Santa Cruz Biotechnology |
| Gt-beads | Glutathione Sepharose^®^ 4B | Amersham Biosciences |
| HRP-secondary antibody | Amersham ECL Mouse IgG HRP-Linked Whole Ab | Merck |
| Imidazole | Imidazole | Sigma-Aldrich |
| Lumigen | Lumigen™ PS-3 substrate | (RPN2132V1, Amersham) |
| Miniprep kit | GenElute^TM^ HP Plasmid Miniprep Kit | Roche |
| *Nhe*I | *Nhe*I Restriction Enzyme | New England Biolabs |
| Ni-NTA | Ni-NTA Agarose | Inviitrogen |
| PS | PS™ Protease | 270843, Amersham |
| *Pvu*II | *Pvu*II Restriction Enzyme | New England Biolabs |
| Polyhistidine-HRP conjugate antibody | Monoclonal antibody | Pierce Biotechnology, Inc |
| Protease inhibitors | Halt Protease Inhibitor Cocktail | 87786, Thermo Scientific |
| Takara DNA polymerase | PrimeSTART™ HS DNA Polymerase | Takara |
| T4 DNA ligase | T4 DNA ligase | New England Biolabs |
| YM10 | Amicon YM10 ultrafiltration system | Millipore |
| PD-10 | PD-10 desalting column | GE Healthcare Bio-Sciences |

# 2. Supplementary Results

**2.1 Construction of the p2GUS multitagged expression system from Precursor 1**

The p2GUS system was engineered from the original Precursor 1 plasmid p‑2GUS‑pr1‑His₁₀‑m*Tfam*(c.1_123del) through a defined four‑step sequential construction strategy designed to incrementally introduce modular affinity and processing elements while preserving the original backbone. This process generated a series of intermediate precursor plasmids (precursors 2–4, Fig. 1 and Supplementary Table S2) and culminated in the final construct p2GUS‑His₁₀‑GST‑PS‑His₁₀‑EK‑m*Tfam*(c.1_123del) (Fig. 1 and Supplementary Fig. S1).

Starting from Precursor 1, the construction pathway yielded the intermediate plasmids p‑2GUS‑pr2‑His₁₀‑m*Tfam*(c.1_123del), p‑2GUS‑pr3‑His₁₀‑PS‑m*Tfam*(c.1_123del), and p‑2GUS‑pr4‑His₁₀‑GST‑PS‑m*Tfam*(c.1_123del), each introducing a specific functional element required for the final multitagged architecture (Figs. 1 and 2).

**Step 1: Generation of a functional *Bgl*II cloning site to obtain precursor 2** In the first step, a functional *Bgl*II cloning site was created in the precursor 2 construct p‑2GUS‑pr2‑His₁₀‑m*Tfam*(c.1_123del). The native *Bam*HI restriction site (ggatcc, positions 359–364) present in Precursor 1 (4731 bp) was replaced with a *Bgl*II site (agatct, positions 362–367) using CiPCR‑based site‑directed mutagenesis with synthetic primers (Fig. 1, Step 1).

Successful generation of the *Bgl*II site was confirmed in plasmids extracted by miniprep (4734 bp) through *Bgl*II restriction digestion (Supplementary Fig S1B) and DNA sequencing (Supplementary Fig S1C).

**Step 2: Introduction of PreScission protease and *Nhe*I sites to obtain precursor 3** In the second step, coding sequences for the PreScission (PS) protease cleavage site and an adjacent *Nhe*I restriction site were introduced into precursor 2 to generate p‑2GUS‑pr3‑His₁₀‑PS‑m*Tfam* (c.1_123del) (Fig. 1, Step 2).

A 301 bp DNA fragment encoding the PS cleavage site and *Nhe*I site was PCR‑amplified using primers RE‑PS/*Nhe*I (Supplementary Table S1), digested with *Bgl*II and *Pvu*II (Supplementary Fig S1D), and ligated into *Bgl*II‑ and *Pvu*II‑digested precursor 2 plasmid (Supplementary Fig S1E). Correct insertion was verified by colony PCR (Supplementary Fig S1F) and sequencing of the final construct (4764 bp), confirming accurate incorporation of the cleavage module (Supplementary Fig S1G).

**Step 3: Insertion of the GST affinity module to obtain precursor 4** In step 3, the glutathione‑S‑transferase (GST) coding sequence (711 bp) was inserted upstream of the PS cleavage site to generate precursor 4 (p‑2GUS‑pr4‑His₁₀‑GST‑PS‑m*Tfam* (c.1_123del; Fig. 1, Step 3).

The GST fragment was PCR‑amplified from pGEX‑6P‑1 using CiPCR‑GST primers (Supplementary Table S1; Supplementary Fig S1H) and inserted by CiPCR into precursor 3 (Supplementary Fig S1I). Following *Dpn*I digestion and *E. coli* transformation, positive clones were identified by colony PCR (Supplementary Fig S1J). Sequencing of purified plasmid DNA (5421 bp) confirmed the correct GST insertion and overall construct integrity (Supplementary Fig S1L).

**Step 4: Introduction of the second His₁₀ tag and enterokinase site to obtain the p2GUS-His_10_-GST-PS-His_10_-EK-m*Tfam*(c.1_123del)** **construct)** In the final step, a second His₁₀ tag followed by an enterokinase (EK) cleavage site was introduced downstream of the GST‑PS module to complete the tandem His₁₀–GST–PS–His₁₀–EK architecture (Fig. 1, Step 4).

Insertion was performed by CiPCR using a chimeric forward primer encoding the His₁₀–EK module and a standard reverse primer (Supplementary Table S1; Supplementary Fig S1M). Due to the asymmetric primer design, insertion efficiency was approximately 50%. Positive transformants were screened sequentially by colony PCR using primer sets CiPCR‑10H/EK1 and CiPCR‑10H/EK2 (Supplementary Fig. S1N and S1O), followed by *Nhe*I restriction analysis of PCR products (Supplementary Fig S1P). Sequencing of purified plasmid DNA (5460 bp) confirmed the successful generation of the final p2GUS construct (Supplementary Fig S1R).

**2.2 Supplementary Figures**

**Supplementary Fig. S1 Experimental generation of the p2GUS-His_10_-GST-PS-His_10_-EK-*mTfam*(c.1_123del) construct from Precursor 1 (p2GUS-pr1-His_10_-*mTfam*(c.1_123del))**

**Supplementary Fig. 1S Experimental generation of the p2GUS-His_10_-GST-PS-His_10_-EK-*mTfam*(c.1_123del) construct from Precursor 1 (p2GUS-pr1-His10-*mTfam*(c.1_123del))**

**Step 1 A** Agarose gel of p2GUS-pr2-His_10_-*mTfam*(c.1_123del) from two independent clones (left and right; ladder, center); **B** *Bgl*II digestion of plasmids in **A** yielding the expected 4734-bp fragment (center and right; ladder, left); **C** electropherograms of p2GUS-pr1-His_10_-*mTfam*(c.1_123del) (top) and p2GUS-pr2-His_10_-*mTfam*(c.1_123del) (bottom).

**Step 2 D** PCR-amplified PS/*Nhe*I fragment, after *Bgl*II/*Pvu*II digestion (right; ladder, left); **E** linearized p2GUS-pr2-His_10_-*mTfam*(c.1_123del) (4461 bp) after *Bgl*II/*Pvu*II digestion (right; ladder, left); **F** colony-PCR with primers RE-PS/*Nhe*I of ligation transformants (ladder in lanes 5 and 13); **G** electropherograms confirming correct PS/*Nhe*I insertion generating p2GUS-pr3-His_10_-PS-*mTfam*(c.1_123del).

**Step 3 H** GST PCR fragment (711 bp, center; ladder, left) and PCR negative control (right); **I** circular p2GUS-pr3-His_10_-PS-*mTfam*(c.1_123del) CiPCR template at the same concentration used I the CiPCR (left; ladder center), CiPCR products (right); **J** colony-PCR of CiPCR transformants with primers CiPCR-GST (ladder, lane 5); **K–L** agarose gel **K** and electropherogram **L** confirming GST insertion into p2GUS-pr4-His_10_-GST-PS-*mTfam*(c.1_123del).

**Step 4 M** CiPCR products from His_10_/EK insertion into p2GUS-pr4-His_10_-GST-PS-*mTfam*(c.1_123del) template (left; ladder center) and circular template (right); **N** Colony-PCR of CiPCR transformants using primers CiPCR-His_10_-EK-1 (Supplementary Table S1) detects colonies lacking the insert, corresponding to CiPCR products amplified by the reverse CiPCR-His_10_-EK primer, which does not introduce the modification; pre-selected negative lanes 5, 6, 10… likely contain the insert (ladder, lane 11). **O** colony-PCR with primers CiPCR-His_10_-EK-2 (Supplementary Table S1) of **N**-pre-selected CiPCR transformants confirming the expected 1670-bp fragment; **P** restriction analysis with *Nhe*I of insert-positive fragments from **O** (uncut); lane 12 shows *Nhe*I-digested positive control from p2GUS-pr4-His_10_-GST-PS-*mTfam*(c.1_123del); **Q** purified plasmid from representative positive clone 6 in **P;** **R** electropherogram confirming correct generation of p2GUS-His_10_-GST-PS-His_10_-EK-*mTfam*(c.1_123del). Ladder sizes (kb) are indicated to the left of each gel.

**3. Uncropped images of Fig 3**

**Uncropped images of Fig 4**
